# The influence of sample temperature on water cluster ion formation for ToF-SIMS studies of frozen hydrated samples

**DOI:** 10.1007/s00216-025-06248-6

**Published:** 2025-12-03

**Authors:** Michael Bäumer, Thorsten Adolphs, Richard E. Peterson, Anoosheh Akbari, Heinrich F. Arlinghaus, Bonnie J. Tyler

**Affiliations:** 1 Institute of Physics, Universität Münster, Wilhelm-Klemm-Straße 10, 48149 Münster, Germany; 2 Center for Soft Nanoscience (SoN), Universität Münster, Busso-Peus-Straße 10, 48149 Münster, Germany

**Keywords:** Frozen hydrated, Water cluster, Temperature, ToF-SIMS, Biofilm, D_2_O

## Abstract

In time-of-flight secondary ion mass spectrometry (ToF-SIMS), analysis of frozen biological specimens at cryogenic temperatures is often necessary to maintain the native 3D structure of the specimen. Frozen hydrated analysis results in interferences from sputtered water cluster peaks that extend over the full spectral mass range. In this study, we have investigated the influence of the analysis temperature from 98 to 183 K on the water cluster spectrum from a frozen hydrated cell-free model biofilm system which contained the antibiotic ciprofloxacin. Below 163 K, the spectrum was dominated by sequences of water cluster ions of the form (H_2_O)_n_X^+^, where X^+^ is either H^+^, NH_4_^+^, or one of at least 16 other small cations. These sequences repeat every 18 mass units. These sequences of water cluster ions begin at m/z 19 and extend to over m/z 2000. Different temperature trends were observed for each cationized water cluster sequence. At a temperature of 153 K, just below the onset of freeze-drying, the (H_2_O)H^+^ cluster signals decline, and many cationized cluster signals go through a local minimum. In this same temperature region, an increase in proton mobility was observed in experiments using D_2_O. The decline in water cluster ion signals at 153 K was accompanied by an increase in the [ciprofloxacin+H]^+^ signal as well as an increase in signals from other organic molecules. Based on these results, 153 K is recommended as the optimum temperature for analysis of ciprofloxacin in frozen hydrated specimens.

**Supplementary Information:**

The online version contains supplementary material available at 10.1007/s00216-025-06248-6.

## Introduction

Time-of-flight secondary ion mass spectrometry (ToF-SIMS) is increasingly important in biological studies because of its capability for label-free 2D and 3D imaging of pharmaceuticals, metabolites, and lipids with sub-micrometer resolution. Because ToF-SIMS measurements must be made under high vacuum, biological samples must either be freeze-dried or frozen and analyzed under cryogenic conditions. Freeze-drying has several drawbacks including migration of analytes, formation of voids, and structural collapse, so cryogenic analysis of frozen hydrated specimens is preferred, particularly for 3D imaging. One drawback to cryogenic analysis of hydrated samples is interference from water cluster ions. Several studies have shown that intense water cluster ion peaks are observed over a wide mass range in ToF-SIMS spectra [1–9]. In a recent study, Akbari et al. used ToF-SIMS to investigate the ciprofloxacin distribution in *Bacillus subtilis* bacteria biofilms. In that study, the water cluster ion peaks interfered with the detection of many common biomolecules and prevented the detection of ciprofloxacin below a concentration of 10 µg/ml [4]. Although the prevalence of water cluster ions in the ToF-SIMS spectra has been previously reported, methods for reducing interference from the water cluster ions have not been systematically studied. In this work, water cluster ions have been investigated using a frozen cell-free model biofilm system containing 1000 µg/ml ciprofloxacin.

Previously, we demonstrated that background signals, caused by the metastable decay of water cluster ions in the time-of-flight analyzer, can be moved relative to stable ion peaks in order to minimize interference with target analytes and avoid misassignment of these metastable signals [9]. However, this strategy only considers excited water clusters, which eject water ions during their flight through the analyzer. Additional strategies must be developed to improve the detection limits for analytes that overlap with stable water cluster ions.

It has been previously shown that the redeposition of water ice on the sample surface can worsen detection limits at low temperatures. Möller et al. found that the redeposition of water ice on a PVC tube decreased significantly between 143 and 163 K [5]. However, the redeposition-free sample temperatures described by Möller et al. are high enough to pose the risk of freeze-drying the sample [6, 7]. With the development of large-gas cluster sputtering (GCIB), it is now possible to remove redeposited water between analysis scans [4]. The aim of this work is to find the optimal analysis temperature below freeze-drying for aqueous sample systems when redeposited ice is removed by GCIB sputtering between each analysis scan.

## Methods

### Sample preparation

Three solutions were prepared:

- sample 1 — A cell-free model biofilm solution (pH 5.08) was prepared containing triply distilled H_2_O (MilliQ) with 150 mM ammonium formate (cryo-protectant), 5 wt. % dextran (surrogate for the extracellular matrix), 1000 µg/ml ciprofloxacin (C_17_H_18_FN_3_O_3_, see figure S1 (top)) and 0.54 % acetic acid (Sigma Aldrich all). Acetic acid was necessary to solubilize the ciprofloxacin.
- sample 2 — Ciprofloxacin in D_2_O solution: A solution identical to solution 1 was prepared, but H_2_O was replaced with D_2_O (99.95%, Deutero GmbH, Germany, CAS: 7789-20-0) to investigate the temperature dependence of protium/deuterium exchanges between D_2_O and ciprofloxacin. The ammonium formate solution contained H_2_O, resulting in a 130:1 ratio of D_2_O to H_2_O.
- sample 3 — A ciprofloxacin-free control, identical to the cell-free model biofilm but without ciprofloxacin, was prepared to investigate the background spectrum and temperature dependence of the water cluster ions.

The solutions were pipetted onto thin copper wells (4 mm in diameter, 100 μm in depth with a 200-μm-thick base plate), plunge-frozen in liquid propane, and then mounted on a pre-cooled copper block under liquid nitrogen.

*Bacillus subtilis* biofilms were grown on specially prepared aluminum planchets which were coated with polydopamine and sterilized in 70% ethanol. An overnight culture of the *B. subtilis* was diluted 1:100 into Lysogeny broth (LB broth) and 300-μl aliquots were transferred to the wells of a 48-well polystyrene microtiter plate (Nunc®) containing the sterile polydopamine-coated aluminum substrates. The biofilms were then allowed to grow in an incubator at 37 °C for 2 to 4 weeks. The medium was changed every 3 days. The biofilm samples were then rinsed in 150 mM ammonium formate and exposed for 5 min to a 1000 µg/ml ciprofloxacin solution in 150 mM ammonium formate. The biofilms were plunge frozen in liquid propane and then mounted on a pre-cooled copper block under liquid nitrogen [4].

The cryo-preparation of all samples was performed in a glove box with relative humidity below 5%. The samples were then transferred to the ToF-SIMS load lock using a Bal-Tec VCT 100 shuttle to minimize frost deposition from exposure to humid laboratory air.

### Data acquisition

ToF-SIMS measurements were made using a custom IONTOF GmbH instrument which is largely equivalent to the M6, which uses a dual-stage reflectron mass analyzer. Prior to the reflectron, the analyzer has two field-free zones which are at different energy potentials. A schematic of the mass analyzer can be found in figure S2. The instrument is equipped with an active liquid nitrogen heating/cooling system in both the load lock and the analysis chamber, which allows precise (± 2 K) temperature control down to 98 K.

All measurements were performed using conditions comparable to those used in earlier 3D imaging experiments on biofilms [4]. The measurements were made in non-interlaced dual beam mode using 0.05 pA and 30 keV Bi_3_^+^ primary ions for analysis and a 20 kV Ar_2000_^+^ for sputtering. The analysis beam was bunched to provide a mass resolution of over 8000 for the [ciprofloxacin+H]^+^ ion (m/z 332.14). A 100 × 100 µm^2^ analysis area and a 500 × 500 µm^2^ GCIB sputter area were used in all measurements. One sputter frame was made for each 128 × 128 pixel analysis frame with a 2-s pause for charge compensation. A 21-V, 2.4-A electron beam was used for charge compensation. Additionally, the analysis chamber was flooded with inert argon to maintain a pressure of 5 × 10^–6^ mbar during analysis. The Ar-flooding assisted with sample charge compensation and minimized pressure fluctuations during the GCIB sputtering of the ice. All measurements were made in positive ion mode using the M6 analyzer in “All Purpose” mode, which employs 2000 V extractor energy and 3000 V analyzer energy (see figure S2).

For analysis of the cell-free model biofilm samples with H_2_O and D_2_O (samples 1 and 2), a GCIB sputter current of 2 nA was used. The analysis temperature was varied from 100 to 183 K by 0.4 K/min. For the ciprofloxacin-free control sample, the sputter current was reduced to 1 nA to allow for a longer analysis time so the sample could be cycled between 123 and 153 K.

The spectra for the biofilm and the cell-free model biofilm were calibrated using (H_2_O)_n_H^+^ peaks (*n* = 1–10) and the [ciprofloxacin+H]^+^ peak. Sample 3 was calibrated using (H_2_O)_n_H^+^ peaks (*n* = 1–10). Calibration on the deuterated solution was performed with (D_2_O)_n_D^+^ peaks (*n* = 2–9) and C^+^ in the lower mass range and [ciprofloxacin-H_2_O+D_3_O]^+^ in the higher mass range.

## Results and discussion

### Spectrum

**Fig. 1:**
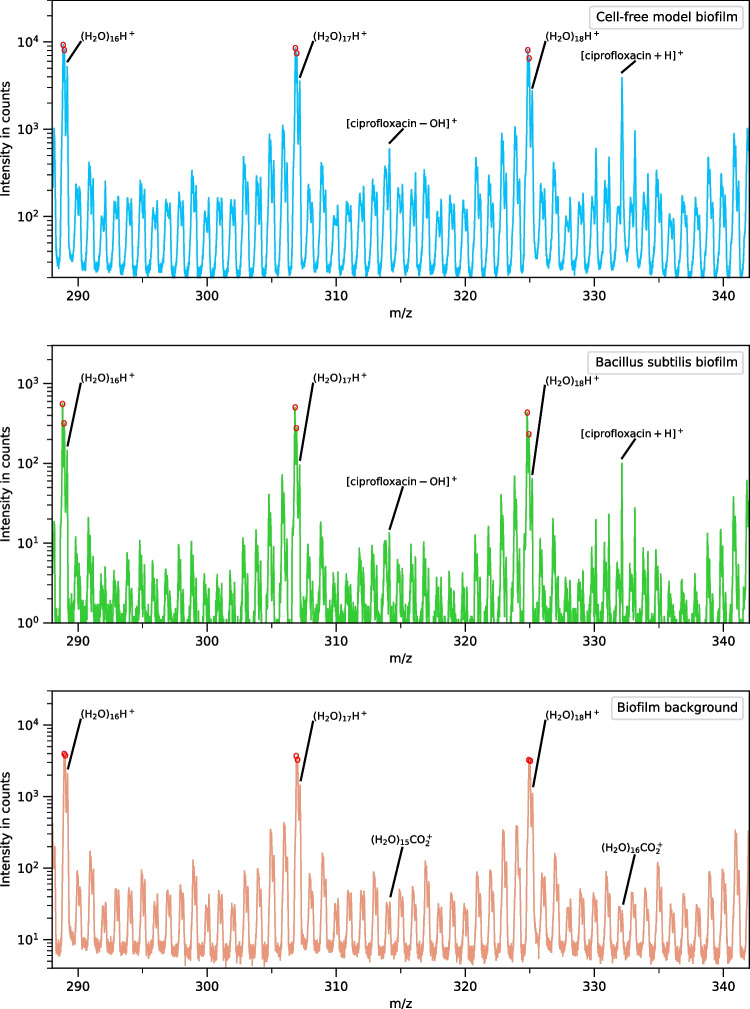
ToF-SIMS spectra in the mass range of m/z 288.5 to m/z 342.5 from the cell-free model biofilm (top), a *B*. *subtilis* biofilm [4] treated with 1000 µg/ml ciprofloxacin (middle) and the ciprofloxacin-free control (bottom). The ciprofloxacin peaks are superimposed on a repeating pattern of cationized water clusters

Figure 1 shows a section of the spectra from m/z 288.5 to m/z 342.5 for the cell-free model biofilm sample 1 (top), a *Bacillus subtilis* biofilm from the study by Akbari et al. [4] (middle), and the ciprofloxacin-free control sample 3 (bottom). In the cell-free model biofilm, the ciprofloxacin and ice are uniformly distributed in the sample (see figure S3). All three spectra show the same pattern of peaks, which arise from water cluster ions. Peaks from ciprofloxacin can be seen in both the cell-free model biofilm and the *B. subtilis* biofilm, superimposed on the repeating water cluster pattern. All other peaks in this range of the spectrum arise from water cluster ions of the form (H2O)_n_X^+^ (henceforth cationized water clusters), where X^+^ is one of at least 18 different cations. In the ciprofloxacin-free control, peaks from CO_2_^+^ cationized water clusters are present at the [ciprofloxacin-OH]^+^ and [ciprofloxacin+H]^+^ masses. The spectra are shown on a log scale to allow better visualization of the smaller cationized water cluster peaks. The largest peaks from stable ions in all three spectra arise from protonated water cluster ions (H_2_O)_n_H^+^, which are labeled in Fig. 1. To the left of each of the protonated water cluster peaks are a pair of metastable peaks, which arise from ejection of a single H_2_O molecule from a higher mass protonated water cluster ion [9–11]. The red dots in Fig. 1 mark metastable peaks generated from the loss of H_2_O from an (H_2_O)_18_H^+^ parent ion. The metastable peaks were unambiguously identified by varying the analyzer voltages. There are two metastable peaks at each nominal mass arising from decays in the two field-free drift zones prior to the reflectron. A detailed description of the behavior of the metastable peaks has been described previously [9]. In the mass range shown, the metastable peaks are more intense than the stable water cluster ion peaks. Between the protonated water cluster ion peaks, there are peaks at every nominal mass. These peaks are from water cluster ions that contain a cation, such as (H_2_O)_16_NH_4_^+^, (H_2_O)_16_CH_3_^+^, and (H_2_O)_15_Na^+^ (plus at least 14 other cations) and peaks from the metastable decay of each of these ions. In ToF-SIMS, analysis of the metastable peaks can be used to unambiguously identify fragmentation pathways and verify molecular structure [11]. The metastable peaks associated with each of these cationized water clusters confirm that they are indeed from water cluster ions (9) (see figure S4 in the Appendix). There are at least 18 different cations in each water cluster sequence. The pattern shown in Fig. 1 between m/z 288.5 and m/z 305.5 is repeated every 18.01 u (the mass of an H_2_O) across the full mass spectrum from m/z 19 through m/z 2000, the highest mass measured.

The [ciprofloxacin+H]^+^ falls within a mass difference of 0.018 u to the (H_2_O)_16_CO_2_^+^ water cluster ion peak, which is unresolvable under the attainable mass resolution. At the high ciprofloxacin concentration used in this study, the (H_2_O)_16_CO_2_^+^ signal is more than two orders of magnitude lower than the [ciprofloxacin+H]^+^ signal, so the interference from the (H_2_O)_16_CO_2_^+^ water cluster is negligible. However, in the study by Akbari et al. [4], the (H_2_O)_16_CO_2_^+^ water cluster peak was found to prevent the detection of ciprofloxacin at concentrations below 10 µg/ml. In that study, the water cluster ions were also found to interfere with many other peaks commonly detected in bacterial biofilms.

A set of measurements was made on a ciprofloxacin-free control with cycled temperature variations. The data (see figure S5) reveal reversible effects below 153 K, so freeze-drying does not occur in the temperature range below 153 K. At a constant temperature of 163 K, the peak intensities of NH_4_^+^ cationized water clusters, smaller molecules, and elements, as well as the analyzer pressure changed significantly. Hence, we suspect that freeze-drying begins several degrees above 153 K, but below 163 K. At temperatures higher than 173 K, rapid freeze-drying is observed. This is consistent with temperature programmed ice measurements by Barros et al. [6, 7] where the ice thickness did not change until temperatures above 157 K.

### The influence of temperature in H2O

**Fig. 2:**
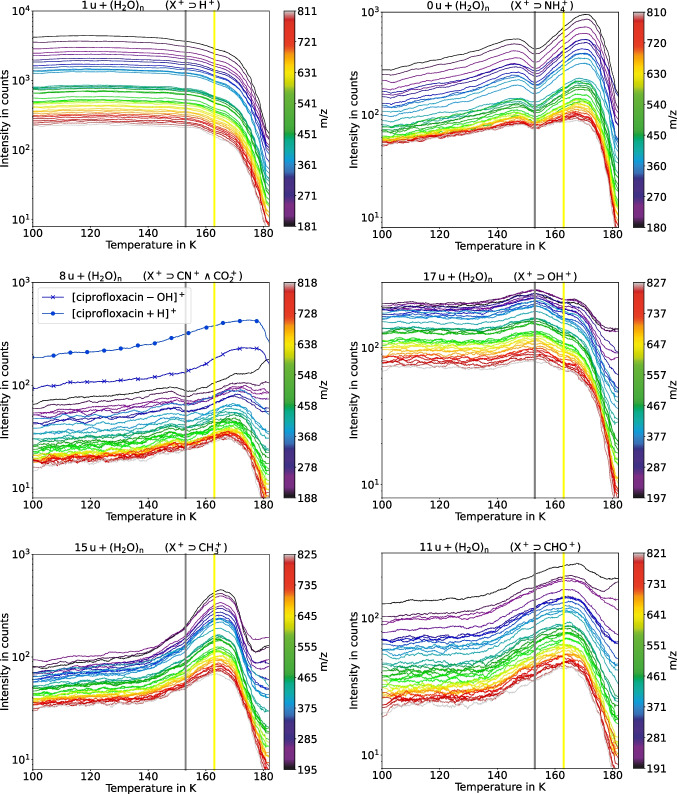
Temperature profile for water clusters with different cations. The line color indicates the mass of the cluster ion as shown in the color bar to right of each figure. Signals from the ciprofloxacin overlap with the CO_2_^+^ cationized water cluster signals. The temperature of 153 K is indicated by the gray line, the temperature of 163 K by the yellow line

The temperature trends for water cluster peaks that contain the same cation were found to be strongly correlated. Figure 2 shows the temperature trends for 6 of these cations for clusters in the mass range from m/z 180 to m/z 827 for the cell-free model biofilm (sample 1). For the protonated water cluster ions, this mass range corresponds to clusters that contain between 10 and 45 water molecules. The data shown in Fig. 2 was binned to 1 u so that metastable and stable peaks at the same nominal mass are combined. Furthermore, temperature trends were smoothed. Temperature trends for the full set of 18 cation masses are provided in the supplemental material figures S6, S7, and S8. The temperature trend for the total ion signal is shown in figure S9. One mass unit above the (H_2_O)_n_ cluster masses, the protonated (X^+ ^⊃ H^+^ means X^+^ is a superset of H^+^) water cluster ion signals (Fig. 2, top left) are at least an order of magnitude higher than the other cationized water cluster ions. The signals from these ions remain approximately constant from 100 until 140 K and then decrease gradually until approximately 173 K when rapid freeze-drying of the sample begins. The intensity gap around 10^3^ counts between the blue and green lines divides the protonated clusters (H_2_O)_21_H^+^ (m/z 379.23) and (H_2_O)_22_H^+^ (m/z 397.24) which are known as a magic and an antimagic water cluster number [12]. The intensity differences are in good accordance with the ones found by Conlan et al. [2]. This gap is an important fingerprint in the mass spectrum of water ice and will be emphasized for this measurement in the supplementary material (see figure S10).

On the same mass at (H_2_O)_n_ cluster masses, the signals from the NH_4_^+^ cationized water cluster ions, the second most intense clusters, show a distinctly different trend (Fig. 2, top right). They rise gradually from 100 to 140 K, drop to a local minimum at 153 K, and then rise to a maximum before the onset of rapid freeze-drying. Eight mass units above the (H_2_O)_n_ cluster masses, the detected signals in the higher mass range are from CO_2_^+^ cationized water clusters (Fig. 2, middle left). In the mass range below m/z 188, CN^+^ cationized water clusters interfere with CO_2_^+^ cationized water clusters. Like the NH_4_^+^ cationized water cluster ions, the CO_2_^+^ cationized water clusters also show a local minimum at 153 K. This is also true for a total of 9 of the 18 cationized water cluster ion sequences (see figures S6, S7, and S8). The same trend is observed for the total ion yield (not shown). In contrast, the OH^+^ cationized water clusters, which are the third most intense, go through a maximum at 153 K and then initially decline gradually, then abruptly above 173 K (Fig. 2, middle right).

The ciprofloxacin peaks, which overlap the CO_2_^+^ cationized water clusters (Fig. 2, middle left, lines are marked with dots and crosses), are not only much more intense than the CO_2_^+^ cationized water clusters, they follow a very different trend with temperature: Ciprofloxacin signals begin to rise around 140 K and continue rising until 173 K and then plummet as the sample rapidly freeze-dries. The water clusters containing CH_3_^+^ (Fig. 2, lower left) or CHO^+^ (Fig. 2, lower right) follow a trend more similar to that of the ciprofloxacin peaks, as do the organic peaks below m/z 100 (data not shown).

**Fig. 3:**
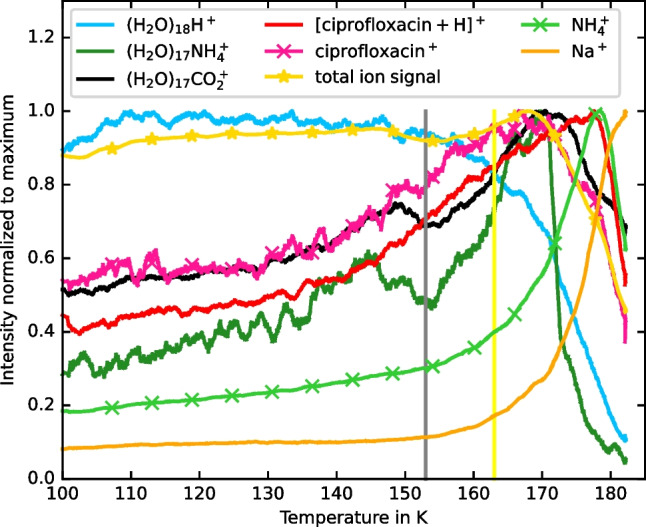
Temperature trends for different cationized water clusters, for protonated and non-protonated ciprofloxacin as well as for NH_4_^+^ and Na^+^ and the total ion signal for Bi_3_^+^ analysis of sample 1. (H_2_O)_n_CO_2_^+^ are combined for the clusters *n* = 10, 11, 12, 13, 17, and 18 in the mass range of ciprofloxacin, but without interference with ciprofloxacin. The temperature of 153 K is indicated by a gray vertical line, the temperature of 163 K by a yellow vertical line

Figure 3 shows an overlay of key stable ion peaks vs. temperature for sample 1 normalized to their maximum. The non-protonated ciprofloxacin signal [C_17_H_18_FN_3_O_3_]^+^ and the protonated ciprofloxacin signal [C_17_H_18_FN_3_O_3_+H]^+^ monotonically increase up to a temperature of 173 K. Above this temperature, rapid freeze-drying occurs and the ciprofloxacin signals decrease rapidly. While the ciprofloxacin signals rise with temperatures, the (H_2_O)_18_H^+^, (H_2_O)_17_NH_4_^+^, and combined (H_2_O)_n_CO_2_^+^ (for *n* = 10, 11, 12, 13, 17, and 18) water cluster signals decline above 140 K. Without risking freeze-drying, an optimum analysis temperature for [ciprofloxacin+H]^+^ occurs at 153 K with a CO_2_^+^ cationized water cluster background reduction of 16–30%. Above the temperature of 153 K, the ciprofloxacin-to-protonated-water-cluster background continues to increase, but the sample begins freeze-drying. The sharp decrease in the ciprofloxacin during rapid freeze-drying suggests that proton transfer from the water ice to the ciprofloxacin enhances the ion yield.

Similar to protonated ciprofloxacin, the signals of (H_2_O)_n_NH_4_^+^ and (H_2_O)_n_CO_2_^+^ increase with increasing temperature above 153 K until rapid freeze-drying occurs. At higher temperatures, freeze-drying results in a sharp rise in NH_4_^+^ and elemental signals (such as Na^+^) which are accompanied by a rise in the signals of the (H_2_O)_n_NH_4_^+^ clusters. The unusual behavior at 153 K, shortly below the freeze-drying temperature, may be due to surface specific melting. Hong et al. observed significant changes in the mechanical properties of water ice surfaces (between 121 and 152 K) with atomic force microscopy (AFM) measurements which indicated a pre-melting of the ice at 152 K. This surface limited pre-melting could facilitate increased mobility of protons and other charge carriers at the surface of the otherwise frozen, solid ice matter [13]. It is likely that the increased proton mobility in the sample allows competition for positive charge between the solutes at 153 K which leads to the local minima of the signals from (H_2_O)_n_NH_4_^+^ and (H_2_O)_n_CO_2_^+^ and the corresponding maximum in the (H_2_O)_n_OH^+^ signal [14, 15].

In order to better understand the unusual behavior of the water signals at 153 K, which is just below the freeze-drying temperature, an additional temperature-controlled ToF-SIMS study was undertaken. A set of measurements was performed on a cell-free model biofilm made with D_2_O (sample 2).

### Comparison of H_2_O and D_2_O sample behavior

**Fig. 4:**
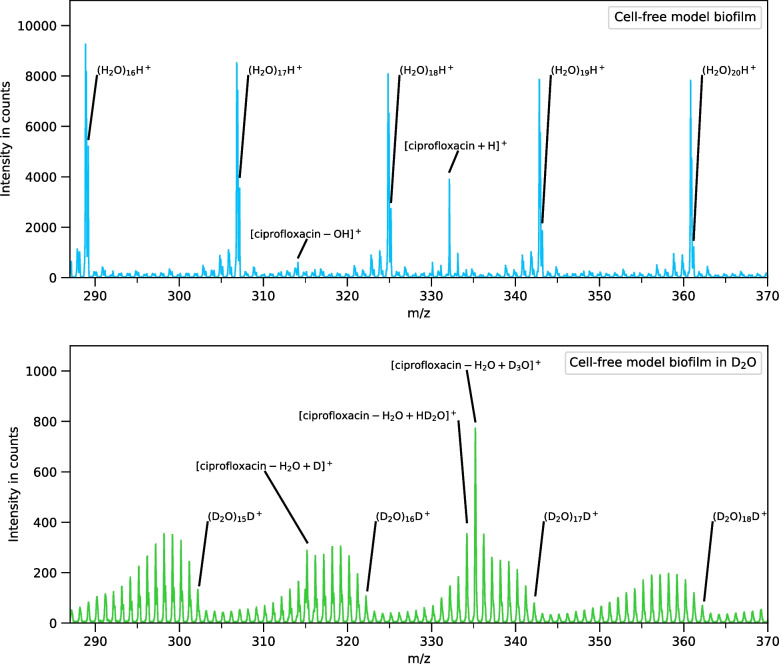
Spectra comparison between hydrated (H_2_O, top) and deuterated (D_2_O, bottom) ciprofloxacin solutions. In hydrated solutions, a series of peaks, separated by 18.01 u, is dominating the spectrum. In impure deuterated solutions, deuterated water clusters have incorporated a different amount of hydrogen atoms and form sequences of water cluster peaks separated by 20.03 u

Figure 4 shows ToF-SIMS spectra from m/z 285 to m/z 375 taken from cell-free model biofilm sample 1 (top), which was made with triply distilled water, and sample 2 (bottom), which was made with D_2_O and contains a 130:1 ratio of D_2_O to H_2_O. In the frozen deuterated sample spectra (bottom), water cluster ion sequences separated by 20.03 u (D_2_O) are observed. Four repeating patterns can be seen between m/z 288 and m/z 370. From m/z 314 to m/z 336, some ciprofloxacin ions superimpose these heavy water cluster ion sequences. Unlike the light water (H_2_O) sample (Fig. 4, top), where the protonated clusters are an order of magnitude more intense than the other cationized clusters, the heavy water (D_2_O) sample (Fig. 4, bottom) shows a repeating pattern of protonated and deuterated water clusters of similar intensity that contain different numbers of protium and deuterium atoms. Protium impure heavy water clusters result from ammonium formate, acetic acid, and ciprofloxacin additives, which have labile hydrogens that can exchange with the initially highly pure D_2_O solvent. The most intense H_x_D_y_O_(x+y)/2_H/D^+^ cluster peaks in the spectrum of the heavy water sample (Fig. 4, bottom) are at least a factor of 20 lower intensity than the (H_2_O)_n_H^+^ cluster peaks seen in the spectrum of the light water sample (Fig. 4, top). For larger deuterated water clusters, the statistical probability for protium-containing clusters increases. Accordingly, higher levels of protium ratios are observed in deuterated water clusters for higher mass ranges. In the heavy water (D_2_O) sample, metastable deuterated water cluster peaks cause strong interference with other cationized deuterated water cluster ions. This prevented further investigation of the cationized water clusters in the deuterated solution (figure S11).

In the heavy water (D_2_O) sample, ciprofloxacin will readily exchange deuterium for the hydrogen atom attached to the piperazine ring and the hydrogen atom attached to the carboxylic acid group, which leads to three distinct ciprofloxacin molecular ions (see figure S1). Although interference with the metastable background associated with the H_5_D_28_O^+^ cluster ion is significant, the signal for the deuterium substituted ciprofloxacin [C_17_H_16_D_2_FN_3_O_3_+D^+^] is clearly identifiable as well as the ciprofloxacin signals at [C_17_H_16_D_2_FN_3_O_3_+H^+^] and [C_17_H_16_D_2_FN_3_O_3_-OD^+^].

**Fig. 5:**
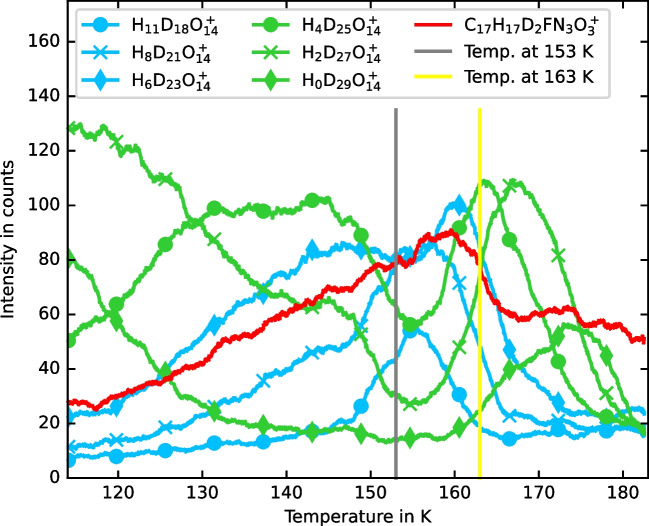
Temperature-dependent peak intensities from a D_2_O water cluster set with 14 oxygen atoms in a mass range without ciprofloxacin fragments. The purest D_2_O cluster (D_2_O)_14_D^+^ is less intense around 155 K (gray vertical line). The most impure shown water cluster H_11_D_18_O_14_^+^ is most intense around 155 K. The temperature of 163 K is indicated by a yellow vertical line

The temperature-dependent intensities of different peaks from a deuterated water cluster sequence with 14 oxygen atoms are shown in Fig. 5. The mass range selected does not include any typical ciprofloxacin fragments. The temperature profiles reveal significantly different temperature trends for clusters depending on the ratio of protium to deuterium. Clusters with a high deuterium-to-protium ratio are most intense at the lowest temperatures. Above 155 K, most water cluster ions observed contain a high protium-to-deuterium ratio. Thus, the exchange of deuterium and protium atoms between D_2_O and organic molecules is pronounced in this temperature region. Above 158 K, the clusters with a high deuterium-to-protium ratio increase, and the clusters with a low deuterium-to-protium ratio decrease.

It is notable that the water clusters with the smaller deuterium-to-protium ratio (Fig. 5, blue) go through a maximum at 155 K, and the clusters with the larger deuterium-to-protium ratio (Fig. 5, green) go through a minimum at this value. A minimum was also observed in NH_4_^+^ cationized water clusters near 153 K in the light water sample (Fig. 2). The slight difference in the optimum temperature between the light and heavy water cell-free biofilm models is consistent with the known differences in phase transition temperatures between light water and heavy water. However, estimating these temperature shifts is challenging, as experimental vapor pressure data in the supercooled regime for D_2_O are still lacking, even in the most recent data [16].

The signal C_17_H_17_D_2_FN_3_O_3_^+^ (protonated ciprofloxacin with two deuterium substitutions) is most intense at 158 K (Fig. 5, red). This supports the hypothesis of enhanced proton mobility near 155 K. However, interference from the metastable H_28_D_9_O_18_^+^ peak with the C_17_H_17_D_2_FN_3_O_3_^+^ signal may also be influencing this trend.

For future studies, the role of a variety of different organic compounds on water cluster formation should be investigated. We propose a ciprofloxacin-like temperature behavior, as similar temperature trends were observed for all organic ions in the low mass range in this work. However, Conlan et al. have shown that different analytes and analyte concentrations shift the equilibrium proton binding in the aqueous environment [1]. The influence of sample pH should also be investigated, as pH values affect proton availability and could alter the influence of temperature on the water cluster ion signals.

Hong et al. used an AFM to investigate water ice surface changes during pre-melting [13]. A combined ToF-SIMS and AFM measurement can deliver insights into changes in ion-bombarded ice surfaces, just before freeze-drying occurs.

Structural changes in cryo-samples can lead to changes in the mechanical properties of the ice surface. Poleunis et al. used Ar-backscattering [17] to observe softening of polymer surfaces during a temperature increase. Softer ice could contribute to higher molecular ciprofloxacin signal intensity by altering the nature of the sputter cascade. Further studies using Ar-backscattering could improve understanding of the changes occurring in the ice.

## Conclusions

ToF-SIMS measurements on frozen hydrated samples reveal a variety of background signals caused by cationized water clusters (such as (H_2_O)_n_H^+^, (H_2_O)_n_NH_4_^+^, and (H_2_O)_n_CO_2_^+^). Temperature variations influence the signal intensities of water clusters and organic analytes. We found that in a frozen cell-free model biofilm, the water cluster background signals that interfere with the antibiotic analyte ciprofloxacin can be reduced by choosing optimal temperature conditions at about 153 K, just below the freeze-drying temperature. At the optimum temperature, the ratio of water cluster peak intensities to analyte was decreased by 16–30%.

In the higher mass range, sequences that repeat every 18.01 u (mass of a water molecule) indicate clusters with a different number of water molecules using the same incorporated cation. Consistent temperature trends were found for water clusters with the same incorporated cations but a different number of water molecules. Signals arising from organic analytes show different temperature trends than the water clusters. Therefore, the investigation of temperature trends can assist with the identification of peaks which are not from water clusters.

The data indicates that proton exchanges are favored around 153 K, which is accompanied by an increase in protonated organic signal intensities, including ciprofloxacin, acetic acid, formic acid, and fragments of dextran. The competition for protons at 153 K inhibits the formation of ammonium cationized water cluster signals as well as the signals from several other cationized water clusters. Measurements in D_2_O also indicated changes in protonation in the same temperature region as H_2_O, but protium-deuterium exchanges were maximized at 155 K.

## Supplementary Information

Below is the link to the electronic supplementary material.

ESM 1 Supplementary Material 1 (DOCX 2.91 MB)

## Data Availability

The data of this study will be made available upon request to Dr. Bonnie J. Tyler.

## Declarations

No biological material was used in this research.
