# Supplementary material for: The influence of sample temperature on water cluster ion formation for ToF-SIMS studies of frozen hydrated samples
Source: Anal Bioanal Chem. 2025 Dec 3;418(3):937–46. doi: 10.1007/s00216-025-06248-6 (PMC12891007; doi:10.1007/s00216-025-06248-6)
Supplement: Supplementary file 1 — Supplementary Material 1 (DOCX 2.91 MB) [file 216_2025_6248_MOESM1_ESM.docx]

Supplementary Information

**The Influence of Sample Temperature on Water Cluster Ion Formation for ToF-SIMS Studies on Frozen Hydrated Samples**

Michael Bäumer^1,2^, Thorsten Adolphs^1,2^, Richard E. Peterson^1^, Anoosheh Akbari^1^, Heinrich F. Arlinghaus^1,2^ and Bonnie J. Tyler*^1,2^

1. Institute of Physics, Universität Münster, Wilhelm-Klemm-Straße 10, 48149 Münster, Germany
2. Center for Soft Nanoscience (SoN), Universität Münster, Busso-Peus-Straße 10, 48149, Münster, Germany

*E-Mail: tyler@uni-muenster.de

Table of Contents

[Figure S1: Ciprofloxacin and potential protium-deuterium exchanges 2](#_Toc213245889)

[Figure S2: M6 ToF analyzer 3](#_Toc213245890)

[Figure S3: Lateral ion distribution in the cell-free model biofilm 4](#_Toc213245891)

[Figure S4: Stable and metastable water cluster pattern 5](#_Toc213245892)

[Figure S5: Temperature trends in the ciprofloxacin free control sample 6](#_Toc213245893)

[Figure S6: Temperature trends for cationized water clusters – part 1 8](#_Toc213245894)

[Figure S7: Temperature trends for cationized water clusters – part 2 9](#_Toc213245895)

[Figure S8: Temperature trends for cationized water clusters – part 3 10](#_Toc213245896)

[Figure S9: Temperature trend for the total ion signal 11](#_Toc213245897)

[Figure S10: Intensity differences in (H_2_O)_21_H^+^ and (H_2_O)_22_H^+^ 12](#_Toc213245898)

[Figure S11: D_2_O Interference 13](#_Toc213245899)

[References 13](#_Toc213245900)

# Figure S1: Ciprofloxacin and potential protium-deuterium exchanges


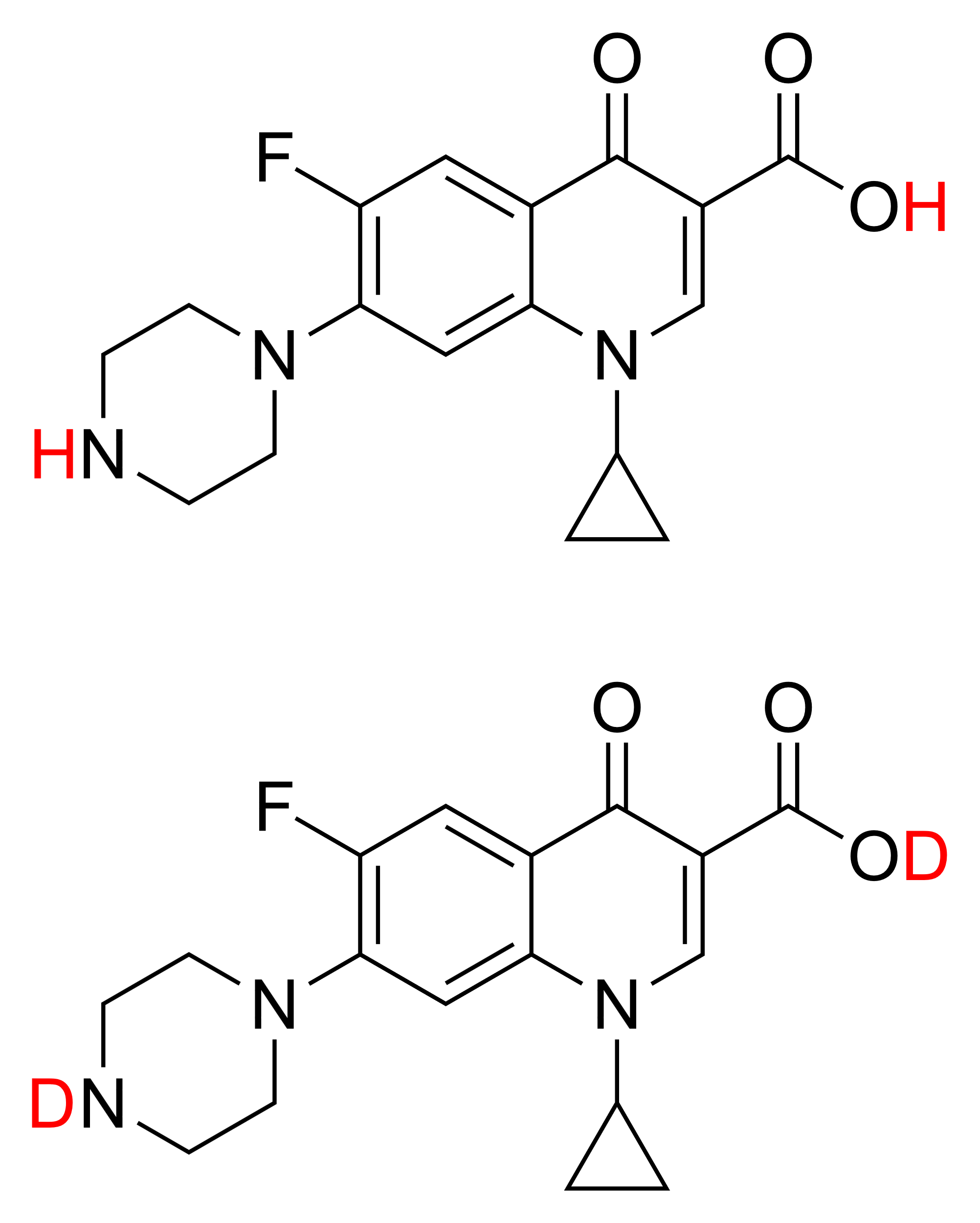


Figure S1: Ciprofloxacin molecule (top) and deuterium substituted ciprofloxacin molecule (bottom).

Figure S1 shows the molecular structure of ciprofloxacin (top). Deuterium exchange is likely for the labile hydrogen atom attached to the piperazine ring (bottom, left) and the labile hydrogen atom attached to the carboxylic acid group (bottom, right).

# Figure S2: M6 ToF analyzer

Figure S2: Scheme (not to scale) of the M6 analyzer (IONTOF GmbH, Münster, Germany). Field-free drift zones are shown in blue, acceleration zones in red. The ions spend about half the flight time in the reflectron (right), including the Variable Drift Path (VDP) which decelerates incoming positive ions by +267 V. The three dotted lines in the reflectron show different trajectories for ions with different reflectron entry energies, such as those present when comparing metastable parent and daughter ions, that decay prior to the reflectron.

# Figure S3: Lateral ion distribution in the cell-free model biofilm

Figure S3: Images of selected ions and total ion signal. Maximum Counts (MC) and Total Counts (TC) are given for each signal. Ciprofloxain and water ice are homogeneously distributed along the sample. Features in the images are due to topogrophy.

# Figure S4: Stable and metastable water cluster pattern

Figure S4: The stable and metastable water clusters show repeatable patterns with a difference of 18.01 u, which equals the mass of a water molecule. The analyte ciprofloxacin clearly disturbs this pattern. There are no other significant analytes which interact with this pattern for masses above m/z 180. It is therefore suggested, that the background above m/z 180 is essentially only described by water clusters and cationized water clusters. This figure was published previously[1] and has been minorly changed to increases visibility for reprinting.

# Figure S5: Temperature trends in the ciprofloxacin free control sample

For analysis of sample 3 (figure S5), a GCIB sputter current of 1 nA was used. The analysis temperature was increased from 123 K to 153 K at a rate of 0.4 K/min. The sample was held at 153 K for 10 minutes and then decreased back to 123 K. A second 0.4 K/min temperature ramp to 163 K was then performed. The sample was then held at 163 K for 10 minutes and then the sample temperature was increased to 176 K.

Figure S5: Temperature programmed measurement of sample 3 (plunge frozen water-solution with ammonium formate, dextran and acetic acid (without ciprofloxacin)). The normalized intensities are averaged over 30 data points to reduce noise. Data points, where a temperature of 153 K is reached, are labeled with a grey area. Temperatures of 163 K are labeled with a yellow area.

Figure S5 shows the intensities of selected signals from the control sample as a function of temperature. Each signal is normalized to its maximum intensity. The sample contains no ciprofloxacin but water (H_2_O), ammonium formate, acetic acid and dextran. As long as the sample temperature was kept between 123 K and 153 K, no visible changes in the ice structure were seen in the instrument’s camera image. While the temperature was held at 153 K for 10 min, the intensity was nearly constant for all peaks observed. Slight clipping of the signal intensities may be originated in temperature inertia as the sample has a slow response time to the changes in temperature programming. It was possible to completely reverse the temperature dependent intensity changes of the peaks by cooling them again to 123 K, thus, frozen samples do not undergo any irreversible processes, such as freeze-drying, under given measurement conditions at temperatures up to 153 K. Any irreversible changes are limited to the outermost layer of the frozen sample and can be removed by GCIB sputtering.

At a constant temperature of 163 K, cationized water clusters (such as (H_2_O)_n_NH_4_^+^, element signals (K^+^, Na^+^ or Cu^+^) and smaller molecules (CHO^+^, NH_3_^+^ and NH_4_^+^), as well as the analyzer pressure changed significantly. This increase is even more pronounced at even higher temperatures under rapid freeze-drying.

# Figure S6: Temperature trends for cationized water clusters – part 1


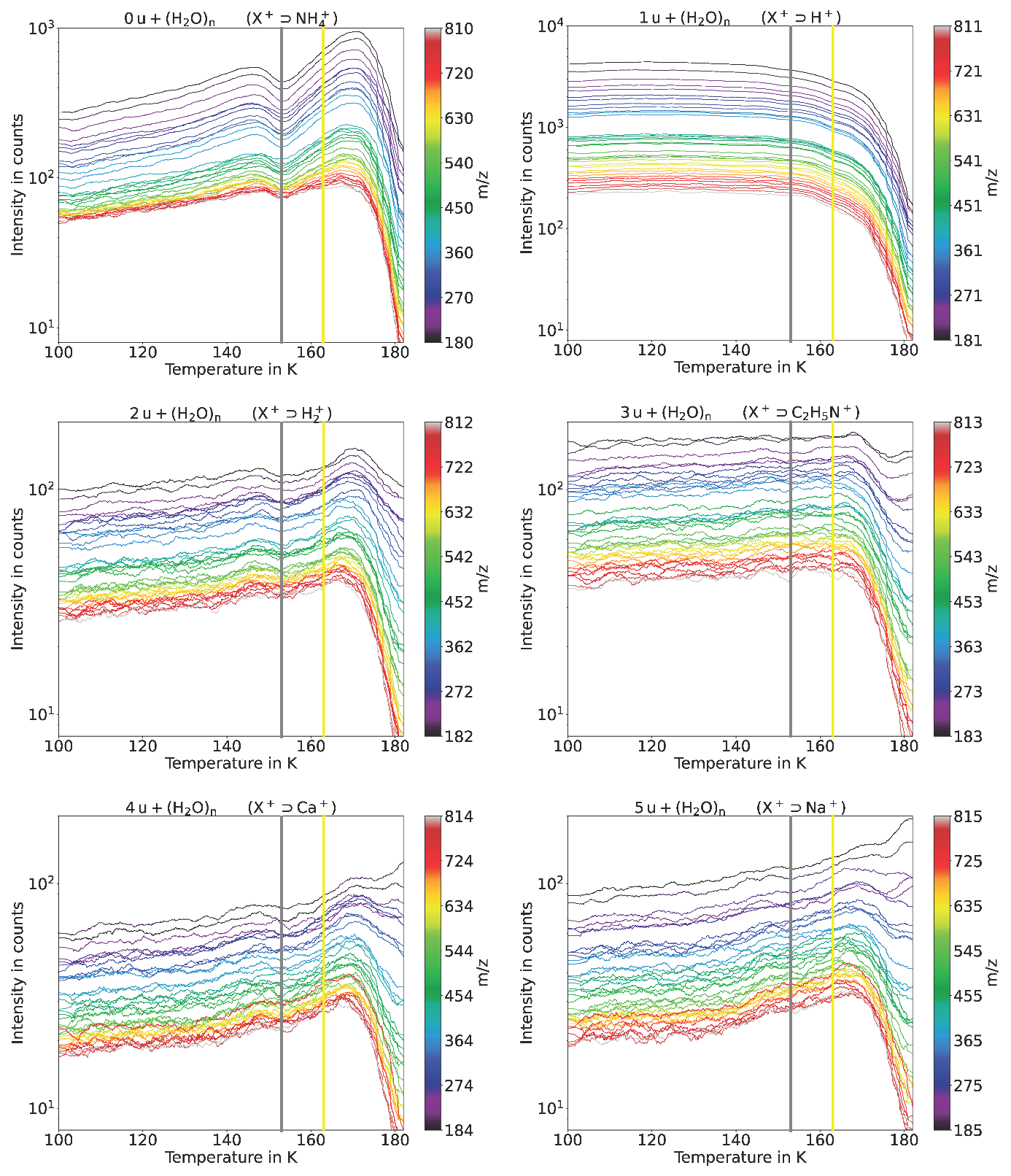


Figure S6: Cationized water clusters (H_2_O)_n_X^+^, where X^+^ is in the mass range of 0 u - 5 u. As the spectrum is superimposed by many organics in the small mass range, only cationized water clusters ≥ m/z 180 are shown. The line color indicates the mass of the cluster ion as shown in the color bar (right). Each subfigure is titled with a possible assignment for X^+^. The assignment is only a subset of the total quantity of possible X^+^ cations. For X^+^ = 0 u, 2 u and 4 u, local intensity minima around 153 K were found. The temperature of 153 K is indicated by a vertical gray line, the temperature of 163 K by a vertical yellow line.

# Figure S7: Temperature trends for cationized water clusters – part 2


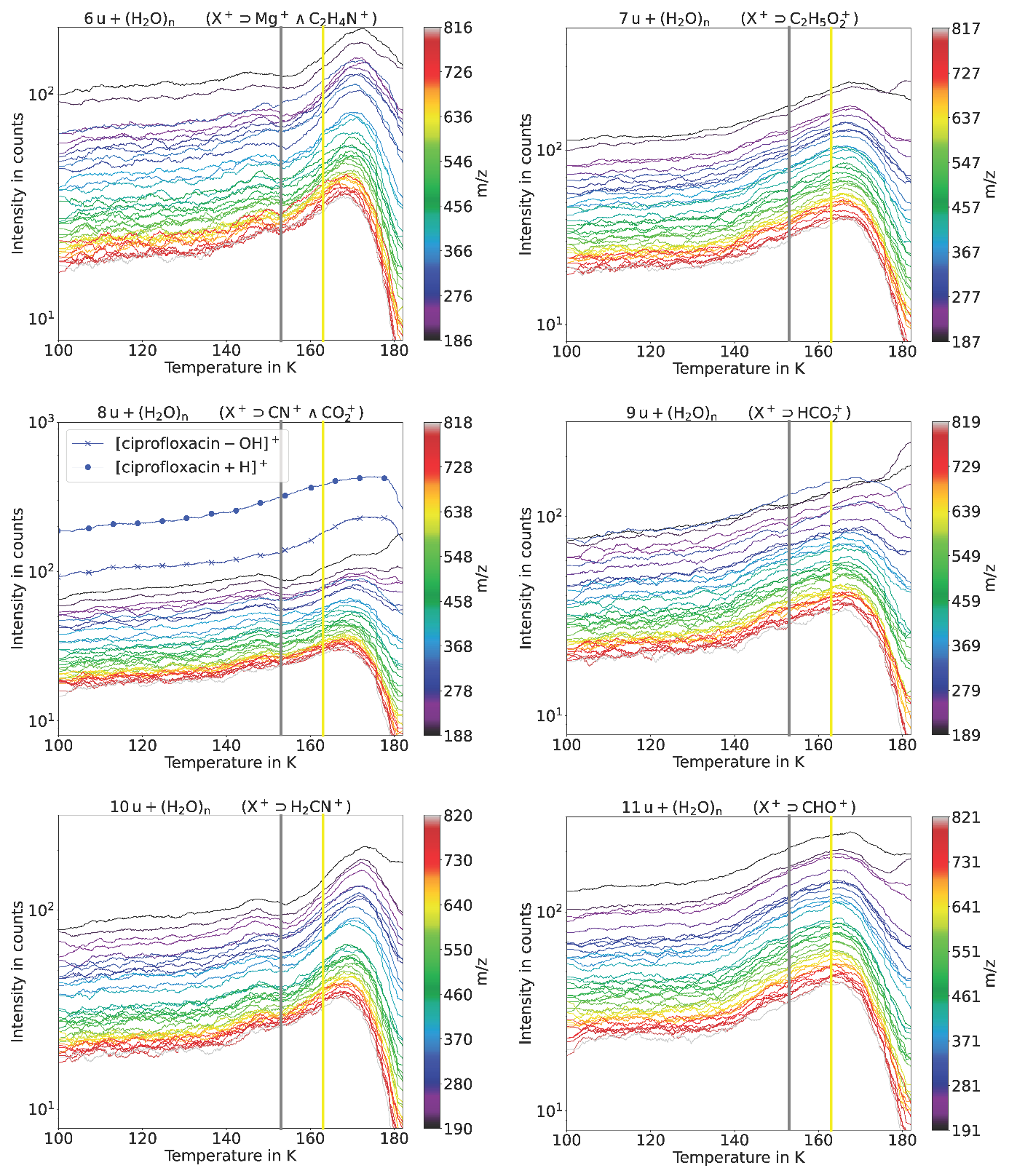


Figure S7: Cationized water clusters (H_2_O)_n_X^+^, where X^+^ is in the mass range of 6 u - 11 u. As the spectrum is superimposed by many organics in the small mass range, only cationized water clusters ≥ m/z 186 are shown. The line color indicates the mass of the cluster ion as shown in the color bar (right). Each subfigure is titled with a possible assignment for X^+^. The assignment is only a subset of the total quantity of possible X^+^ cations. For X^+^ = 6 u, 8 u and 10 u, local intensity minima around 153 K were found. The temperature of 153 K is indicated by a vertical gray line, the temperature of 163 K by a vertical yellow line.

# Figure S8: Temperature trends for cationized water clusters – part 3


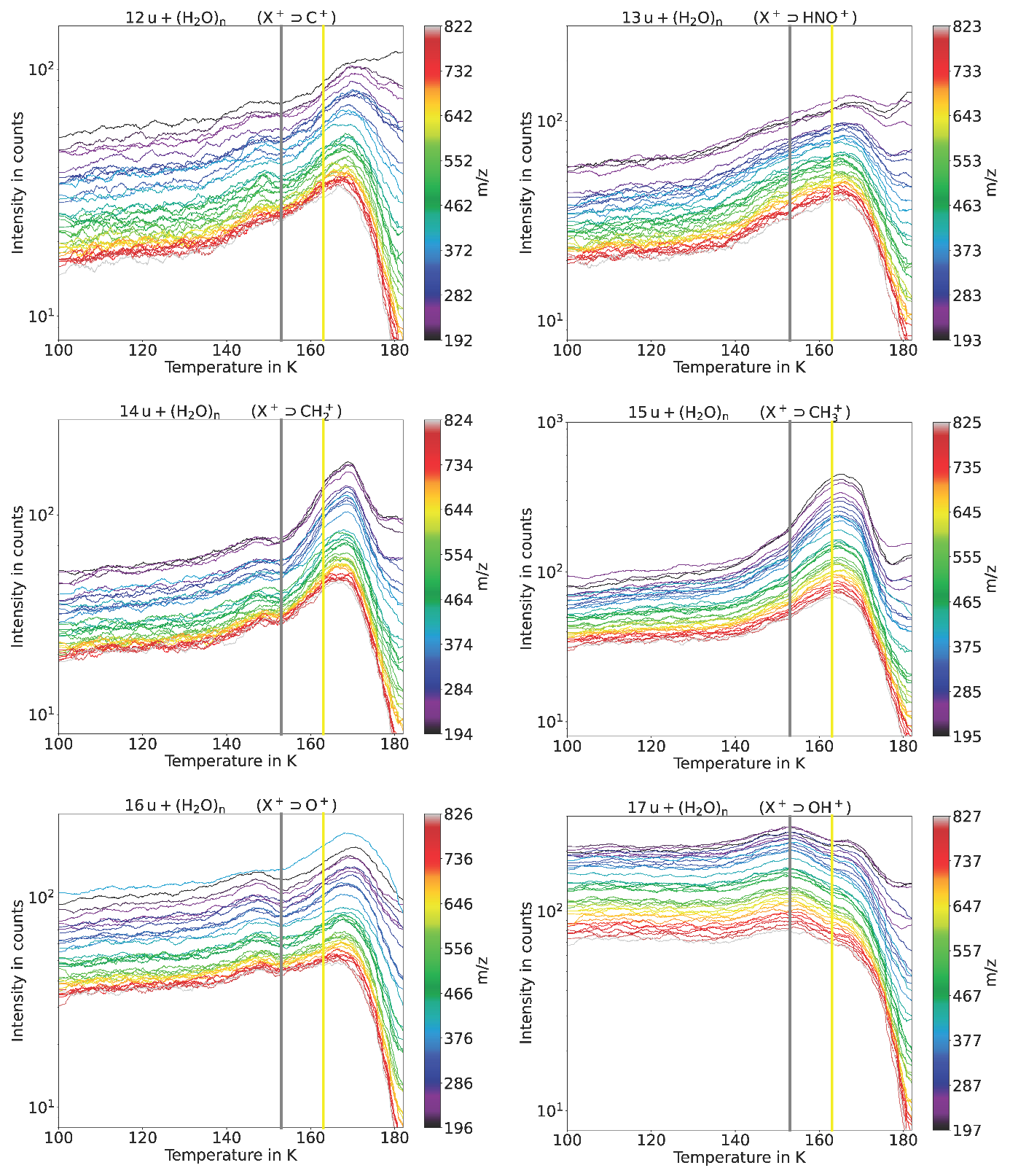


Figure S8: Cationized water clusters (H_2_O)_n_X^+^, where X^+^ is in the mass range of 12 u - 17 u. As the spectrum is superimposed by many organics in the small mass range, only cationized water clusters ≥ m/z 192 are shown. The line color indicates the mass of the cluster ion as shown in the color bar (right). Each subfigure is titled with a possible assignment for X^+^. The assignment is only a subset of the total quantity of possible X^+^ cations. For X^+^ = 12 u, 14 u and 16 u, local intensity minima around 153 K were found. The temperature of 153 K is indicated by a vertical gray line, the temperature of 163 K by a vertical yellow line.

# Figure S9: Temperature trend for the total ion signal

Figure S9: Temperature trend for the total ion signal. The temperature of 153 K is indicated by a vertical gray line, the temperature of 163 K by a vertical yellow line.

# Figure S10: Intensity differences in (H_2_O)_21_H^+^ and (H_2_O)_22_H^+^

Figure S10: Intensity differences between (H_2_O)_21_H^+^ and (H_2_O)_22_H^+^. The area of the stable clusters (black) show a ratio of (H_2_O)_21_H^+^/(H_2_O)_22_H^+^ = 4.26, which is in good accordance to the intensity differences measured by Conlan et al.[2]. In SIMS, sputtered water clusters enter the analyzer far from thermal equilibrium which reduces the number of large stable clusters significantly. The area of the stable clusters (especially (H_2_O)_22_H^+^) is clearly influenced by noise. The metastable daughter peak at lower apparent masses (green) shows a ratio of (H_2_O)_21_H^+^/(H_2_O)_22_H^+^ = 1.52 and the metastable daughter peak at higher apparent masses (blue) shows a ratio of (H_2_O)_21_H^+^/(H_2_O)_22_H^+^ = 4.01. The ratio over the whole mass ranges shown (1 u) are (H_2_O)_21_H^+^/(H_2_O)_22_H^+^ = 1.77, which is directly responsible for the gap shown in figure 2 (top left).

# Figure S11: D_2_O Interference


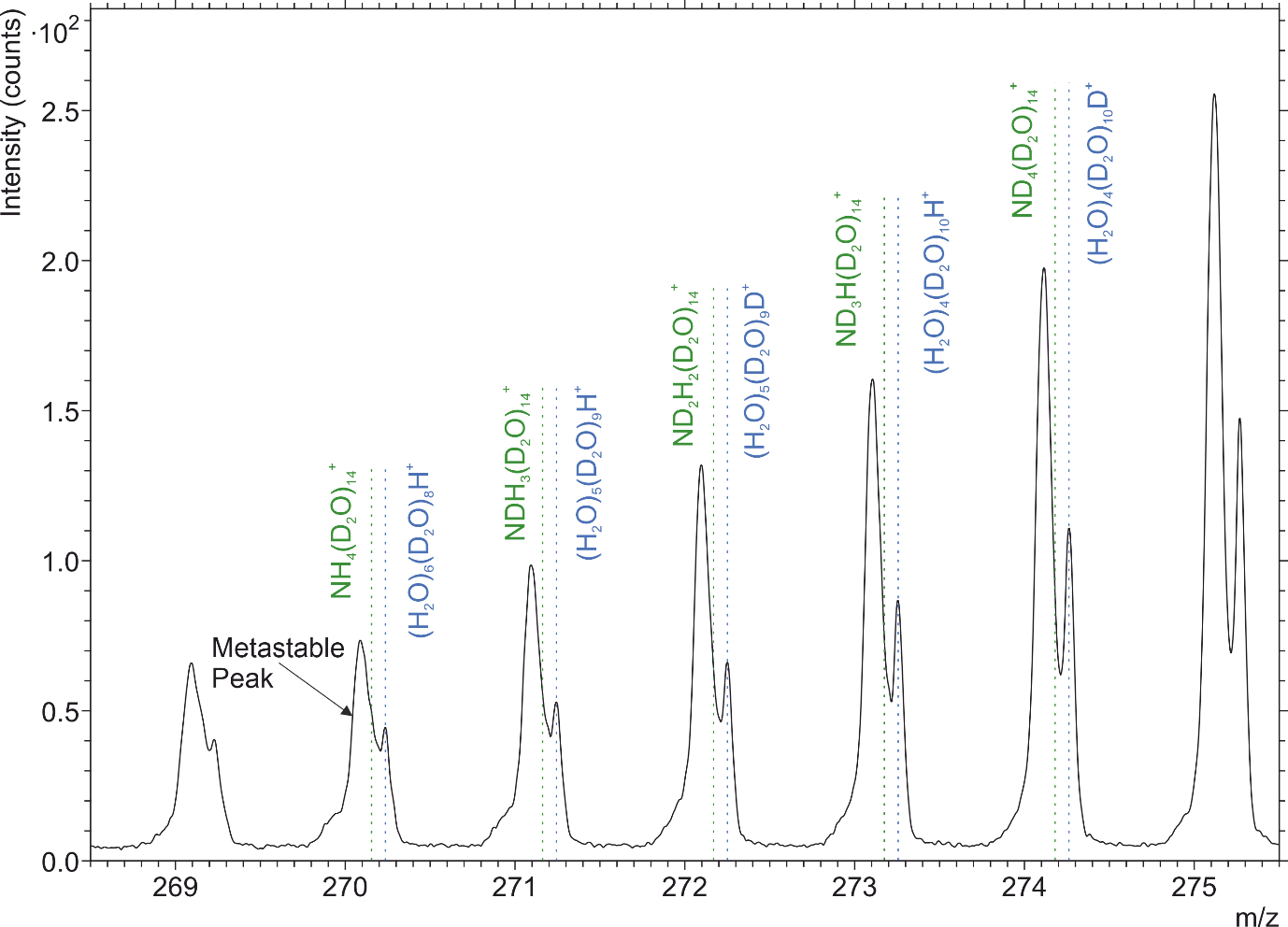


Figure S11: Interference between H/D^+^ and N(H/D)_4_^+^ cationized water clusters and metastable background in the impure D_2_O solution (sample 2).

Figure S11 shows the positions of several partially deuterated water cluster ions. There metastable peaks interfere with other partially deuterated NH_4_^+^ cationized water clusters. This prevents further investigation of the cationized water clusters and several organics in the deuterated solution.

# References

1. Bäumer M, Adolphs T, Peterson RE, Arlinghaus HF, Tyler BJ (2025) Strategies for Minimizing Interference from Metastable Water Clusters in ToF-SIMS 3D Imaging of Frozen Hydrated Biological Samples. Journal of the American Society for Mass Spectrometry. doi:10.1021/jasms.5c00251

2. Conlan XA, Fletcher JS, Lockyer NP, Vickerman JC (2010) A Comparative Study of Secondary Ion Emission from Water Ice under Ion Bombardment by Au+, Au3+, and C60+. The Journal of Physical Chemistry C 114 (12):5468-5479. doi:10.1021/jp906030x
